# Corns

**Published:** 1856-01

**Authors:** 


### CORNS

Are nature's barricades, the skin hardens itself in order to afford protection to the inner and more delicate parts against an ill-fitting shoe, for whether too tight or too loose, the result is similar.

Corns are the deserved punishments of all pretenders and make believes. You endeavored when younger, to persuade people to think that your understandings were less extensive than they really were, *illæ hinc lacrymæ;* hence those outbursts of passion which invaded corns daily give rise to; how instinctively is fended off, the tread of youth and beauty even, by the gallant beau of forty five. What unpleasant reminiscences of our infirmities, are these self same corns in dull weather, the very time when we need some extra exhilaration. A man whom I did not know from Adam came into my office yesterday, Nov. 14th, sat down *vis-a-vis*, took me by the hand, "*I'll read you through, from infancy up*," said he.

"Read away," said I.

I fixed my eyes on the ceiling, he his on the carpet for a "spell," as Jonathan would say.

"What a kindly nature," was his abrupt exclamation, "kind towards everybody and everything, generous to a fault, decidedly frank, too much so for your own good, free, you were born in Kentucky, in one of the inland counties, where there were many girded trees standing, in a log house, not two stories high, and more than one, the upper windows were smaller than those below, I see a door with a window on one side, and perhaps on the other, with casings around them, a temporary shed at one end of the house, the right, while to the left and back, the ground trended rapidly away," &c.—But what in the world has this to do with corns on people's toes? It was pertinent enough, if we had stopped at the right place, but our pen editorial, is like two things, first, like the man with a cork leg, it worked so well, that when he got once started, he couldn't stop, so he has been going ever since; shouldn't wonder if he had got the other side of the moon by this time; second, like that *member* of scripture classics, belonging to one of our household.

"*What a kindly nature*!" we should have stopped there, and given an illustration, in the fact of our being so benevolent, as to publish to our subscriber world, a cure for corns, infallible, *for nothing!*

Never let anything harder than your finger nail ever touch a corn; paring it, as certainly makes it take deeper root, as cutting a weed off at the surface. The worst kind of corns are *controllable*, as follows:

Soak the feet in quite warm water for half an hour before going to bed, then rub on the corn with your finger for several minutes, some common sweet oil. Do this every night; and every morning, repeat this rubbing in of oil with the finger, bind on the toe during the day, two or three thicknesses of buckskin, with a hole in the centre to receive the corn; in less than a week, in ordinary cases, if the corn does not fall out, you can pinch it out with the finger nail; and weeks, and sometimes months will pass away, before you will be reminded that you had a corn, when you can repeat the process. Corns, like consumption, are never cured, but may be indefinitely postponed. The oil and soaking softens and loosens the corn, while the buckskin protects it from pressure, which makes it perhaps to be pushed out, by the under growth of the parts.

As to the log cabin, which had the honor of first sheltering us on the beautiful moonshiny night, Tuesday, October Twenty third, Eighteen Hundred and—so forth—among the hours of the morning, we must write for information to our revered sire, who still has much of the energy of us, his son, and if we can manage to bring it in smooth like, we may report in some subsequent number.

